# Genome mining for methanobactins

**DOI:** 10.1186/1741-7007-11-17

**Published:** 2013-02-26

**Authors:** Grace E Kenney, Amy C Rosenzweig

**Affiliations:** 1 Departments of Molecular Biosciences and of Chemistry, Northwestern University, Evanston, IL 60208, USA

**Keywords:** methanobactin, methanotroph, particulate methane monooxygenase, copper, chalkophore, TonB-dependent transporter, natural product, post-translational modification

## Abstract

**Background:**

Methanobactins (Mbns) are a family of copper-binding natural products involved in copper uptake by methanotrophic bacteria. The few Mbns that have been structurally characterized feature copper coordination by two nitrogen-containing heterocycles next to thioamide groups embedded in a peptidic backbone of varying composition. Mbns are proposed to derive from post-translational modification of ribosomally synthesized peptides, but only a few genes encoding potential precursor peptides have been identified. Moreover, the relevance of neighboring genes in these genomes has been unclear.

**Results:**

The potential for Mbn production in a wider range of bacterial species was assessed by mining microbial genomes. Operons encoding Mbn-like precursor peptides, MbnAs, were identified in 16 new species, including both methanotrophs and, surprisingly, non-methanotrophs. Along with MbnA, the core of the operon is formed by two putative biosynthetic genes denoted *MbnB *and *MbnC*. The species can be divided into five groups on the basis of their MbnA and MbnB sequences and their operon compositions. Additional biosynthetic proteins, including aminotransferases, sulfotransferases and flavin adenine dinucleotide (FAD)-dependent oxidoreductases were also identified in some families. Beyond biosynthetic machinery, a conserved set of transporters was identified, including MATE multidrug exporters and TonB-dependent transporters. Additional proteins of interest include a di-heme cytochrome *c *peroxidase and a partner protein, the roles of which remain a mystery.

**Conclusions:**

This study indicates that Mbn-like compounds may be more widespread than previously thought, but are not present in all methanotrophs. This distribution of species suggests a broader role in metal homeostasis. These data provide a link between precursor peptide sequence and Mbn structure, facilitating predictions of new Mbn structures and supporting a post-translational modification biosynthetic pathway. In addition, testable models for Mbn transport and for methanotrophic copper regulation have emerged. Given the unusual modifications observed in Mbns characterized thus far, understanding the roles of the putative biosynthetic proteins is likely to reveal novel pathways and chemistry.

## Background

Methanotrophs are Gram-negative bacteria that use methane, a potent greenhouse gas, as their sole source of carbon and energy [1]. As the only biological methane sink, methanotrophs have attracted much attention as a means of mitigating methane emissions [2-4]. The first step in their metabolic pathway, the oxidation of methane to methanol, is catalyzed by methane monooxygenase (MMO) enzymes, which are of broad interest in the quest to exploit abundant natural gas reserves as fuel and chemical feedstocks. Most methanotrophs utilize particulate methane monooxygenase (pMMO), a copper-dependent integral membrane enzyme [5,6]. Under copper-limiting growth conditions, some methanotrophs can also express an alternative, soluble form of MMO (sMMO) that utilizes iron [7]. In these methanotroph strains, the switch between pMMO and sMMO is controlled by copper: copper represses transcription of the sMMO genes and causes formation of intracytoplasmic membranes that house pMMO [8-10]. The details of this "copper switch" regulatory mechanism are not understood and represent a major outstanding question in the field.

An important part of the copper switch puzzle is the discovery of methanobactins (Mbns), a family of copper-binding natural products initially detected in the methanotroph *Methylosinus trichosporium *OB3b [11-13], and potentially useful in applications ranging from wastewater copper removal in the semiconductor industry [14] to treatment of Wilson disease, a human disorder of copper metabolism [15]. Mbns are believed to be secreted under copper limiting conditions in a copper-free (apo) form to acquire copper from the environment and then internalized in a copper-loaded form to provide essential copper to the methanotroph [16,17]. In support of this model, methanobactin (Mbn) promotes the copper switch [18,19] and can mediate release of copper from insoluble mineral sources [19,20]. In addition, direct uptake of copper-loaded Mbn (CuMbn) by *Methylosinus trichosporium *OB3b has been demonstrated, and proceeds via an active transport process [21]. Because this model for Mbn function as well as aspects of its structure (*vide infra*) are reminiscent of iron siderophores, Mbn has also been referred to as a chalkophore [13] (*chalko*- is derived from the Greek word for copper whereas *sidero*- is from the Greek word for iron).

**Figure 1:**
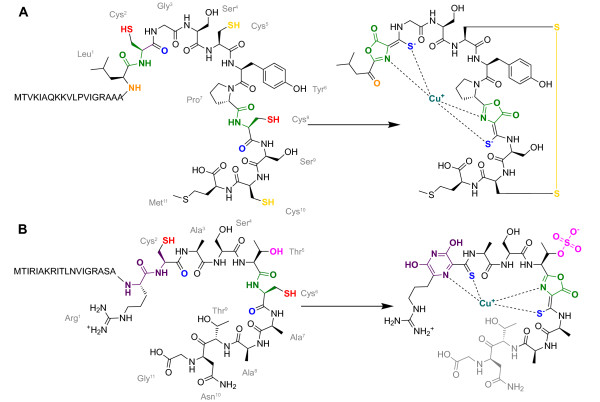
**Post-translational modifications required to produce methanobactins from *Methylosinus trichosporium *OB3b and *Methylocystis rosea *SV97T**. (**A**) Mbn from *Methylosinus trichosporium *OB3b is generated from the precursor peptide MTVKIAQKKVLPVIGRAAALCGSCYPCSCM. Post-translational modifications needed to produce the final natural product include leader peptide cleavage and subsequent N-terminal transamination (orange), oxazolone formation (green), thioamide formation (blue), and disulfide bond formation (yellow). (**B**) Mbn from *Methylocystis rosea *SV97T is generated from the precursor peptide MTIRIAKRITLNVIGRASARCASTCAATNG. Post-translational modifications needed to produce the final natural product include leader peptide cleavage, pyrazinedione formation (purple) oxazolone formation (green), thioamide formation (blue), and threonine sulfonation (pink). Several residues that are present in the precursor peptide are missing in the reported structure (gray); the loss of a C-terminal threonine and asparagine had been previously reported, but identification of the precursor peptide indicates that a final glycine is also lost.

Mbn molecules from *Methylosinus trichosporium *OB3b, *Methylocystis *strain SB2, *Methylocystis hirsuta *CSC-1, *Methylocystis *strain M and *Methylocystis rosea *SV97T have been characterized by mass spectrometry, nuclear magnetic resonance (NMR) and crystallography (Figure 1; Additional file 1, Figure S1). These data reveal a peptidic backbone and copper coordination by two nitrogen-containing heterocycles next to thioamide groups [13,22-25]. The *Methylosinus trichosporium *OB3b Mbn backbone has the sequence 1-(N-(mercapto-(5-oxo-2-(3-methylbutanoyl)-oxazol-(Z)-4-ylidene)methyl)-Gly^1^-Ser^2^-Cys^3^-Tyr^4^)-pyrrolidin-2-yl-(mercapto-(5-oxo-oxazol-(Z)-4-ylidene)methyl)-Ser^5^-Cys^6^-Met^7 ^and is thought to derive from the peptide backbone LCGSCYPCSCM (Figure 1A).

By comparison, *Methylocystis *Mbns are alanine-rich, and the first nitrogen-containing heterocycle is not an oxazolone [24]. All the *Methylocystis *Mbns have a similar backbone. The N-terminal residue is either arginine- or methionine-derived (the latter only in *Methylocystis hirsuta *CSC-1), and immediately precedes the first heterocycle. The heterocycle/thioamide pair (pyrazinadione in all structures except the NMR structure of *Methylocystis *strain SB2) is followed by an alanine, a serine and a sulfonated threonine (Additional file 1, Figure S1). Next is an oxaozlone/thioamide pair and an alanine followed by a methionine (*Methylocystis *sp. M) or a second alanine (Additional file 1, Figure S1). Additional C-terminal residues are present in some forms of the molecule. The *Methylocystis rosea *SV97T Mbn contains a Thr-Asn sequence [24], and likely derives from a peptide backbone containing the sequence RCASTCAATN (Figure 1B). Despite these structural differences, these Mbns retain their strong and specific affinity for copper [24].

The Mbn biosynthetic pathway has not been elucidated, and was initially suggested to involve nonribosomal peptide synthetases [16,26], similar to production of many siderophores [27]. However, sequencing of the *Methylosinus trichosporium *OB3b genome [28] led to the identification of a 30-amino acid open-reading frame (ORF) with similarities to the peptidic Mbn backbone [23], supporting previous suggestions that Mbn is produced via post-translational modification of a ribosomally synthesized precursor peptide [22]. A similar precursor peptide was identified in an unrelated species, *Azospirillum *sp. B510, along with several conserved neighboring genes [17,22], but analogous ORFs were not detected in other available methanotroph genomes, and the relevance of many of the neighboring genes surrounding the precursors was unclear.

Genes encoding the precursors of small ribosomally-produced natural products can be difficult to detect and annotate, and the underdetection of biologically relevant small ORFs is a known problem [29-31]. However, the ever-increasing rate at which bacterial genomes are released has prompted the design of genome mining tools for widespread classes of ribosomally synthesized and post-translationally modified peptide natural products (RiPPs), such as lantibiotics [32-37]. With the aim of identifying the potential for Mbn production in a wider range of bacterial species, we mined the available microbial genomes in the National Center for Biotechnology Information (NCBI) and Joint Genome Institute (JGI)/Integrated Microbial Genomes (IMG) databases, identifying 18 new Mbn-like precursors and accompanying biosynthetic genes from 16 species, including unknown or provisionally identified species present in metagenomic samples. Surprisingly, many of these precursor peptides and their operons are from non-methanotrophic species and several well-studied methanotrophic species seem to lack Mbn operons similar to that of *Methylosinus trichosporium *OB3b. Beyond biosynthesis-related genes, we also identified a widely-conserved set of transporters and sigma factors, which has implications for Mbn export and import as well as its involvement in cellular copper homeostasis. Finally, this bioinformatics study provides new tools to better detect Mbn-like gene clusters in novel genomes.

## Results and discussion

**Figure 2:**
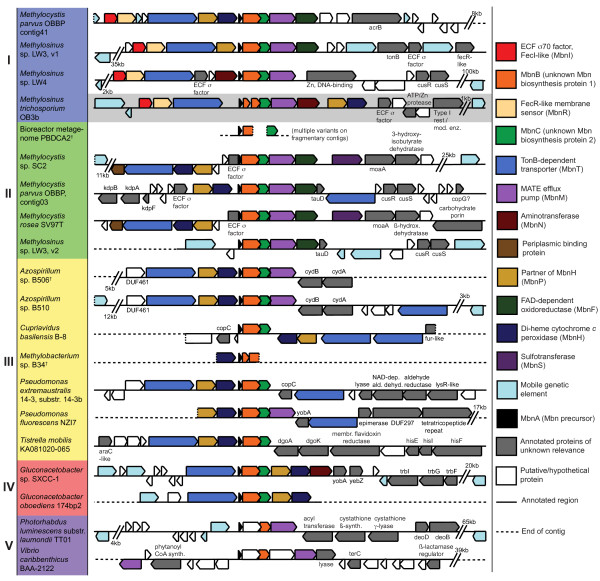
**The genetic organization of Mbn-producing operons**. Genomic regions containing all identified Mbn operons are depicted. Operons are grouped into five families on the basis of operon content, MbnB conservation, and MbnA sequence. All operons contain MbnA (black) and MbnB (orange) and at least one transport protein, a MATE efflux pump (purple), a TonB-dependent transporter (blue) or both. Most operons contain additional biosynthesis-related genes, including MbnC (green), aminotransferases (MbnN, brown) or sulfotransferases (MbnS, dark purple). Additional genes may be related to regulation (MbnR, yellow and MbnI, red) or may play an unknown role in copper homeostasis (MbnP, gold and MbnH, dark blue) Genetic mobility elements of several varieties (light teal) are common in the vicinity of these operons. Metagenomic samples corresponding to unidentified or provisionally identified species are marked (†).

Using a variety of bioinformatics techniques, we were able to detect putative biosynthesis operons for Mbn-like natural products in 14 new species, as well as several unidentified or tentatively identified species present in metagenomic studies (Figure 2; Additional file 2, Table S1). While five of the identified species are Type II methanotrophs like the first identified Mbn-producer, *Methylosinus trichosporium *OB3b, the remaining species are not. Operons were detected in β- and γ-proteobacteria as well as α-proteobacteria, to which the Type II methanotrophs belong. Both the precursor peptides and the range of non-core biosynthesis genes present in the operon hint at a set of potential modifications that may define the Mbn family. Furthermore, genes likely to be related to export, import and copper regulation are found in almost every operon. Based on sequence analysis, the presence of specific Mbn-related genes and the overall operon structure, we have provisionally divided the operons into five groups (Figure 2).

### Locating the precursor peptide MbnA

Automated detection of small peptide sequences in newly-sequenced genomes is problematic [29]. Short sequences are poorly detected by Basic Local Alignment Search Tool (BLAST) and similar sequence analysis methods, and uncurated small ORF detection results in the annotation of many spurious small ORFs. For well-established classes of small ribosomally-produced natural products, such as bacteriocins, hidden Markov model (HMM)-based tools, such as BAGEL and BAGEL2, have been developed to better detect precursors in newly sequenced genomes [32,33]. With only two published precursor peptide sequences (MbnAs), Mbn was not a good candidate for this detection method [17,23]. A TIGRFAM group (TIGR04071) does exist for the precursor, and is a member of the GenProp0962 family (which also includes TIGRFAM groups for MbnC and half of MbnB) [38], but it is based on only the two previously published precursor peptide sequences and a third suggested MbnA homologue from *Gluconacetobacter *sp. SXCC-1 [38]. Four possible MbnAs detected here are also mentioned in the 2013 TIGRFAM update [38], but are not included in the available HMM.

Because of the limitations of direct precursor peptide detection, we pursued an alternate genome mining strategy focusing on the detection of biosynthetic proteins, followed by manual identification of unannotated precursors. This method has been used with some success for a variety of natural products, including radical S-adenosyl methionine (SAM)-modified peptides, bacteriocins in cyanobacteria, and a new class of lantibiotic-like natural products stemming from nitrile hydratase or Nif11 leader peptides [34-36,39]. We used the MbnB and MbnC sequences from *Methylosinus trichosporium *OB3b [38] as seeds in a tBLASTn search through the NCBI's Non-redundant (NR) and Whole Genome Shotgun (WGS) databases, as well as the microbial genomes available at JGI/IMG. For every MbnB homologue detected, a 2 kb region preceding and following that gene was manually examined for 45 to 150 bp ORFs coding for short peptides with at least one cysteine in the last 10 amino acids and an N-terminal region containing multiple arginine or lysine residues.

**Figure 3:**
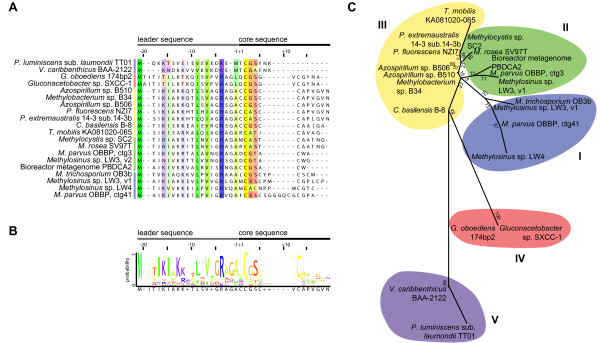
**Alignment and classification of the precursor peptide MbnA**. (**A**) A MAFFT alignment of the 20 sequenced MbnA precursors organized by family. Likely leader and core peptides are indicated on the basis of the structurally characterized Mbns and their corresponding MbnA sequences. The alignment is visualized in Jalview and residues are colored following the Taylor color scheme [109], with intensity modified by a conservation color increment of 30%. (**B**) Logo showing that both leader peptide and core peptide exhibit well-conserved motifs, but the leader peptide is less variant. (**C**) Phylogenetic tree (constructed in MEGA 5.0 as described in the Methods section) based on a ClustalOmega alignment of the 20 full MbnA. Not pictured in this tree are the three species with structurally characterized Mbns for which no genomic information exists; however, their core peptide sequences indicate that they would be grouped with *Methylocystis *sp. SC2 and *Methylocystis rosea *SV97T. Gamma distribution parameter 4.0462.

A total of 18 novel *MbnA*-like ORFs were identified using these methods, one preceding every close *MbnB *homologue excluding truncated homologues from metagenomic sequencing. Two *Methylosinus *species (*Methylosinus *sp. LW3 and *Methylocystis parvus *OBBP, which may be misclassified as *Methylocystis *[40]) have two distinct MbnA genes encoding unique Mbns. While it is not uncommon for bacteria to produce multiple siderophores to control iron acquisition in different environments [41], a similar phenomenon has not yet been observed for chalkophores. As shown in the Multiple Alignment with Fast Fourier Transform (MAFFT) alignment (Figure 3A), both the leader and core sequences exhibit some conservation over the 20 complete sequences. MbnA-like sequences range from 23 to 35 amino acids (aa), with predicted core sequences ranging from 7 to 15 aa. The leader peptides are better conserved than the core peptides, perhaps indicating the involvement of the leader peptide in interactions with biosynthesis proteins [42]. The leader sequences are lysine/arginine rich, with at least two such residues occurring near the beginning and one present in a conserved area immediately prior to the core sequence (Figure 3B). The core sequences are more variable, but all contain at least one C (G|A|S) (S|T) motif. Of the complete MbnA sequences, 18 have a second core cysteine and 11 contain one or two additional cysteines.

One basis for the proposed five operon groups (Figure 3C) is the nature of the MbnA sequences, including the structurally, but not genomically, characterized *Methylocystis *Mbns. The Group I MbnA sequences, primarily from *Methylosinus *genera are long (11 to 15 aa), with four non-adjacent core peptide cysteines, and contain core prolines. It is unknown whether the presence of four cysteines allows for the formation of disulfide bonds as found in *Methylosinus trichosporium *OB3b Mbn or whether they lead to the production of additional oxazole/thioamide pairs, analogous to the multiple thiazoles and oxazoles present in many bacteriocins [42].

The primarily *Methylocystis *Group II MbnA sequences are shorter, contain only two or three cysteines, and many have a conserved threonine which, based on NMR and crystal structures [23,24], is likely to be a sulfotransferase target. Interestingly, the sequences from *Methylocystis *strain SC2 and *Methylocystis rosea *SV97T appear to be merged with an extracytoplasmic function (ECF) sigma factor, at least based on the annotation [43]. It is not clear whether the precursor peptide is cleaved from these sigma factors and whether sigma factor activity remains or is altered. Although there is no structure for Mbn from *Methylocystis *strain SC2, its MbnA sequence and the similarity of its operon structure to that of *Methylocystis rosea *SV97T suggest that its Mbn will resemble *Methylocystis rosea *SV97T Mbn and will be identical to *Methylocysis hirsuta *CSC-1 Mbn [24]. Similarly, although there are no genomes for *Methylocystis *strain SB2, *Methylocystis *strain M and *Methylocystis hirsuta *CSC-1, we can predict that the core peptides for their structurally characterized Mbns will be RCASTCAA, RCASTCAMT and MCASTCAAT, respectively (likely followed by -TNG, -NG and -NG), and that their leader sequences will resemble those from *Methylocystis rosea *SV97T and *Methylocystis *strain SC2 [24,43]. A subfamily of Group II MbnAs from *Methylosinus *or related species (*Methylosinus *sp. LW3, *Methylocystis parvus *OBBP and a bioreactor metagenome) do not have the CASTCA(A) motif. Instead, the second cysteine is followed by a tryptophan. If the core peptide sequence dictates cysteine modification, these residues lack the C(G|A|S) motif associated with cyclization and thioamide formation in existing Mbn structures.

The remaining families include MbnAs from a variety of non-methanotrophic species. The species that have Group III MbnA sequences include two *Pseudomonas *species, two *Azospirillum *species and single species each from the *Cupriavidus*, *Tistrella *and *Methylobacterium *genera. In this group, the two *Pseudomonas *sequences, the two *Azospirillum *sequences and the *Methylobacterium *sequence are most similar, with somewhat lengthy and near-identical MbnA core sequences containing two cysteine doublets. The less similar *Cupriavidus basilensis *B-8 MbnA preserves the cysteine doublets whereas the *Tistrella mobilis *KA081020-065 sequence contains only two non-adjacent cysteines.

The Group IV MbnA sequences are currently only found in the two *Gluconacetobacter *species. These two sequences are nearly identical, and feature only two core cysteines, with a leader sequence potentially extended by two amino acids. Finally, the Group V MbnA-like sequences are found in *Vibrio caribbenthicus *ATCC BAA-2122 and *Phorhabdus luminescens *subsp. *laumondii *TT01. These sequences are short and somewhat divergent, containing only a single cysteine, which may suggest that they represent a natural product with some structural similarities to Mbn that either does not chelate copper or does not chelate copper in the same way that other chalkophores do. This overall classification scheme extends to the MbnB and MbnC sequences (*vide infra*), and will be subject to future modification as more MbnA sequences are identified in new genomes.

### The first unknown biosynthesis protein: MbnB

**Figure 4:**
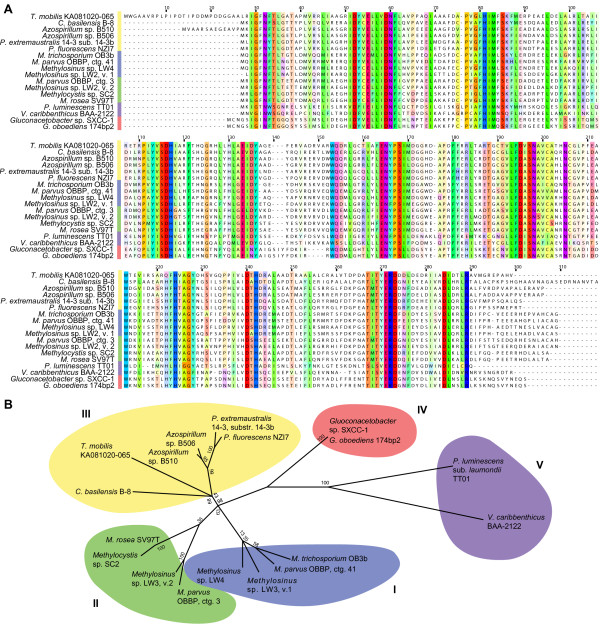
**MbnB: the core Mbn biosynthesis gene**. (**A**) MUSCLE alignment of MbnB, including both domains. Residues are colored following the Taylor color scheme [109], with intensity modified by a conservation color increment of 30%. Truncated metagenomic sequences are not shown in this alignment. (**B**) Phylogenetic tree (constructed in MEGA 5.0 as described in the Methods section) based on a MUSCLE alignment of all MbnB sequences, not including truncated metagenomic sequences. Gamma distribution parameter 1.5823.

MbnB is the core protein in the Mbn biosynthesis operon, and was detected in 19 operons, including truncated forms in several metagenomic samples (Figures 2 and 4; Additional file 2, Table S1). However, the initial identification of this protein in *Methylosinus trichosporium *OB3b has been problematic. In *Methylosinus trichosporium *OB3b, and one other operon detected (*Methylobacterium *sp. B34), MbnB is split into two ORFs, formerly annotated as MettrDRAFT_3894 and MettrDRAFT_3895, but reannotated as one entity (MettrDRAFT_3422) in a recently assembled genome build available on IMG [23]. A TIGRFAM HMM (TIGR04159) exists for the half of the protein that resembles MettrDRAFT_3894, but does not cover MettrDRAFT_3895 [38]. Therefore, a conjugate with a glycine replacing the stop codon between the two ORFs was used for BLAST detection and annotation.

Despite the addition of new members to the MbnB family, no motifs or domains of known function have been identified beyond occasional classification as TIM-barrel proteins [44]. MbnB homologues may be a subfamily in the larger DUF692 family (PFAM class PF05114). However, when conducting a BLAST search or a HMM-based search for homologues, MbnB-like proteins represent a distinct subgroup, with a sharp drop-off in expectation value between the last MbnB-like protein (E <1E-50, except for sequences truncated by the end of a contig/scaffold) and other DUF692-like proteins. Notably, in the Group V operons, the *MbnB *gene is separated from the MbnA-like precursor by a gene-sized ORF.

A comparison of MbnB sequences (Figure 4A) strongly supports the five operon families assigned on the basis of the MbnA sequences (Figure 4B). There are about six different regions that are strikingly well-preserved, even in the Group V homologues. Without knowledge of the structure or function of MbnB, it is difficult to interpret which of these conserved regions are important. However, given that MbnB and MbnC are the only proteins with unassigned functions that are preserved in both the *Methylosinus trichosporium *OB3b and *Methylocystis rosea *SV97T operons, it is possible that one or both are responsible for the nitrogen-containing heterocycles and the neighboring thioamides that have been present in every Mbn structure obtained thus far.

### The second unknown biosynthesis protein: MbnC

**Figure 5:**
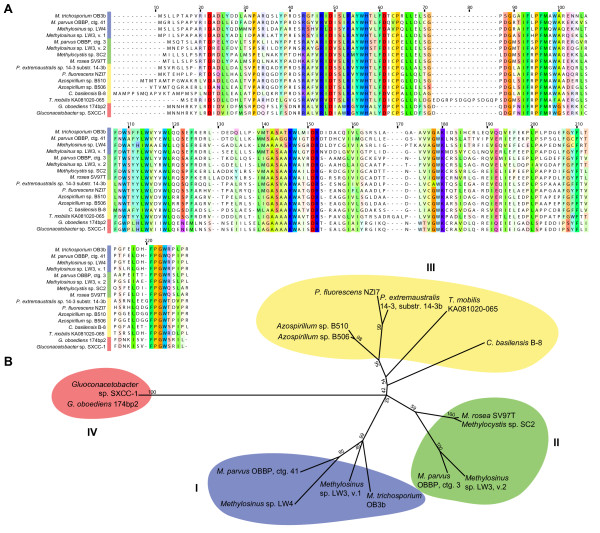
**MbnC: the secondary Mbn biosynthesis gene**. (**A**) MUSCLE alignment of MbnC putative biosynthesis proteins. The Group V sequences are divergent and not included. Residues are colored following the Taylor color scheme [110], with intensity modified by a conservation color increment of 30%. Truncated metagenomic sequences are not shown in this alignment. (**B**) Phylogenetic tree (constructed in MEGA 5.0 as described in the Methods section) based on a MUSCLE alignment of all MbnC sequences, not including truncated metagenomic sequences. Gamma distribution parameter 2.1637.

MbnC is the second unknown Mbn biosynthesis protein, and as with MbnB, there is an existing, if limited, TIGRFAM class (TIGR04061) [38]. We detected MbnC-like proteins in 17 novel operons, a number that includes two fragmentary hits in a bioreactor metagenome (Figure 5A). As with MbnB, there is a broader class of distantly related hits (with high *Pseudomonas *representation and a more divergent C-terminal region), visible after a sharp decline in expectation value quality. This set of more distant relatives appears to correspond to the TIGRFAM family TIGR04061.

MbnC homologues are present in Groups I to IV Mbn operons. For those operons, the phylogenetic tree constructed for MbnC resembles that for MbnB and MbnA, supporting the proposed classification scheme (Figure 5B). In families with a true MbnC homologue, the predicted MbnC ORF frequently overlaps MbnB by a significant number of residues, but it is not in frame with MbnB. As with MbnB, multiple alignment of MbnC homologues confirms the broad conservation of several regions of the gene, but the relationship between the conserved regions and MbnC's potential role in biosynthesis of thioamide and nitrogen-containing heterocycles remains unclear.

The Group V operons, which appear to be the most distantly related to the *Methylosinus trichosporium *OB3b operon, diverge with MbnC. There do not appear to be clear Group V homologues for MbnC as there are for MbnB. There is, however, an unidentified ORF immediately neighboring the precursor, conserved primarily in these two species. This ORF could possibly encode a core biosynthetic protein for the Group V operons (Additional file 1, Figure S2). These sequences appear to have no close homologues in other species, and have a weak N-terminal similarity to the DUF692-like domain (PF05114), which is more like MbnB than MbnC.

### Other biosynthesis proteins: MbnN, MbnS and MbnF

The Mbns from *Methylosinus *and *Methylocystis *species exhibit post-translational modifications beyond the formation of nitrogen-containing heterocycles and neighboring thioamides. Mbn biosynthesis in *Methylosinus trichosporium *OB3b requires a transamination reaction on the N-terminal amine group of the core peptide following leader peptide removal, as well as the formation of a disulfide bond, and all four *Methylocystis *Mbns contain a sulfonated threonine group [22,24]. Although specific proteases and disulfide-forming proteins are not evident, we have discovered proteins likely responsible for transamination and threonine sulfonation in the Mbn biosynthesis operons of several genomes. Transaminases are present in three operons only: *Methylosinus trichosporium *OB3b (annotated as "histidinol phosphate transaminase/cobyric acid decarboxylase" and with a PFAM classification of PF00155 or Class I/II aminotransferase), *Methylosinus *sp. LW4 (also PF00155 or class I/II aminotransferase), and *Gluconacetobacter *sp. SXCC-1 (classified as PF00202 or Class III aminotransferase) (Figure 2). The transaminase has tentatively been designated MbnN. The paucity of transaminases in Mbn operons suggests that the N-terminal transamination present in *Methylosinus trichosporium *OB3b Mbn may not be a common modification.

Like the N-terminal transamination, threonine sulfonation may only be present in a subset of Mbns. To date, it has only been observed in the four structures of Mbns produced by *Methylocystis *species [23,24]. Sulfotransferases with domains corresponding to Pfam family PF00685 were detected only in the two Group II *Methylocystis *operons. Although no structure for Mbn from *Methylocystis *strain SC2 is available, the similarity of its MbnA to that of *Methylocystis rosea *SV97T combined with the presence of a sulfotransferase in its operon strongly suggests that its Mbn will also be sulfonated, presumably at the same threonine. This sulfotransferase has been designated MbnS.

Finally, the gene encoding MbnF, generally annotated as a flavin adenine dinucleotide (FAD)-dependent monooxygenase or an FAD-dependent oxidoreductase (Pfam PF01494), is also present in six Group I and II operons (including all known *Methylocystis *genomes and some *Methylosinus *genomes), always following MbnM (Figure 2). The function of MbnF is unclear, but given its presence in the *Methylocystis rosea *SV97T operon and absence in the *Methylosinus trichosporium *OB3b operon, it could play a role in pyrazinedione biosynthesis (Figure 1), possibly hydroxylating the heterocycle. Without structures of Mbn-like products from non-methanotrophs, it is difficult to connect other neighboring genes (annotated or not) to potential biosynthetic modifications and to determine the effective ending point of the operon and potentially the end of any multicistronic mRNA transcripts. In both *Methylocystis *species, *MbnS *is followed by a gene resembling *MoaA*, a protein responsible for the first step in molybdenum cofactor biosynthesis [45] (which involves the conversion of a guanosine derivative to precursor Z) and a gene generally annotated as a 3-hydroxyisobutyrate dehydrogenase. Hypothetical unknown proteins (including the *MbnC *replacement in Group V operons) are present in several operons, and a range of proteins of unknown relevance, including several varieties of known copper-related proteins, appear in a few operons only (Figure 2).

### Exporting methanobactin via MbnM

A proton/sodium-dependent multidrug export pump (MATE), belonging to the PFAM class PF01554, is found in 13 of the identified operons (Figure 2). Of the remaining operons, several are on small contigs in more fragmented draft genomes making it difficult to rule out the presence of a similar exporter. Excluding *Vibrio caribbenthicus *and *Photorhabdus luminescens*, which appear to have dissimilar MATE transporters, perhaps reflecting a less similar final Mbn-like product, this exporter is well-conserved, even in the non-methanotrophs *Pseudomonas fluorescens *NZI7 and *Azospirillum *sp. B510 and B506 (Additional file 1, Figure S3.) In prokaryotes, MATE transporters primarily function as exporters of antibiotics and similar toxic compounds, simultaneously importing Na^+ ^or H^+ ^and exporting mostly cationic natural products [46-48]. Native natural products are primarily exported by non-MATE efflux pumps, such as the resistance-nodulation-cell division (RND) or major facilitator superfamily (MFS) exporters that are believed to transport some siderophores out of the cell [49-51]. However, many MATE transporters do not have known substrates, and MATE transporters are even found in antibiotic hypersensitive strains [52]. Thus, the ability of a MATE transporter to secrete Mbn-like compounds is plausible, if unprecedented.

### Importing copper-loaded methanobactin via MbnT

A family of small molecule importers, known as TonB-dependent transporters (TBDTs), are also commonly associated with the Mbn biosynthesis operons. The only genomes for which nearby TBDTs are not observed are *Vibrio caribbenthicus *and *Photorhabdus luminescens*, as well as the second Mbn operon in *Methylosinus *sp. LW3, which is small and surrounded by transposon elements; contig truncation of several other operons may be hiding additional potential transporters in other species. We have shown previously that CuMbn is imported via an active process [17,21] and TBDTs are good candidates for importers since they play a similar role for siderophores [53-56]. TBDTs found in the vicinity of Mbn operons are generally annotated as siderophore receptors and classifiable under models including TIGR01783 (full siderophore-specific TBDT model), PF00593 (TBDT barrel only), PF07715 (TBDT plug domain only) and in some cases PF07660 (an extended N-terminal region, which appears to approximate the published N-terminal extension (NExT) domain [57]); they have provisionally have been designated the MbnT family. Conservation of these TBDTs is weaker than that of MbnB, MbnC or MbnM; even the plug domain displays less homology (Additional file 1 Figure S4A, B). However, differences in the core peptide backbone sequence may require markedly different binding approaches. While methanotroph Mbn-related genes are generally relatively similar, the plug domain sequences of *Methylocystis *Group II TBDTs and *Methylosinus *Group I TBDTs diverge markedly, perhaps reflecting the structural differences of the final compounds (Additional file 1, Figure S4A, B.)

### MbnT may have a FecIRA-like regulation system in *Methylosinus *species

In four operons from *Methylosinus *species, the TBDT has an extra N-terminal domain (Additional file 1, Figure S4C.) These larger TBDTs are preceded by an ORF generally annotated as an "Fe(III) dicitrate membrane sensor" (PFAM PF04773) and an "ECF sigma factor" (with conserved σ-70-like regions 2 (PFAM PF04542) and 4 (PFAM PF08281)), designated *MbnR *and *MbnI*, respectively (Figure 2). This pairing is generally observed for FecIRA-like systems, in which the holo siderophore-bound TBDT interacts with the membrane sensor, which then interacts with the ECF sigma factor to regulate expression of siderophore biosynthesis and transport proteins [57-60]. The earliest example of this system is the eponymous FecIRA system, which controls the transcription of iron citrate transporters [57,59,61,62]. Similar systems exist for siderophores, such as pseudobactins BN7 and BN8 (the PupBRI system) [63], pyoverdines (FpvARI/PvdS) [64] and a range of other siderophores. Not all of these systems have identical regulatory pathways. The pyoverdine transport system has two ECF sigma factors (FpvI and PvdS) which regulate different operons [64], and the HasISR system, which transports heme, has an unusual regulatory scheme in which the membrane-bound sigma factor HasS inhibits the activity of the ECF sigma factor HasI until heme binding to the TBDT HasR [65].

**Figure 6:**
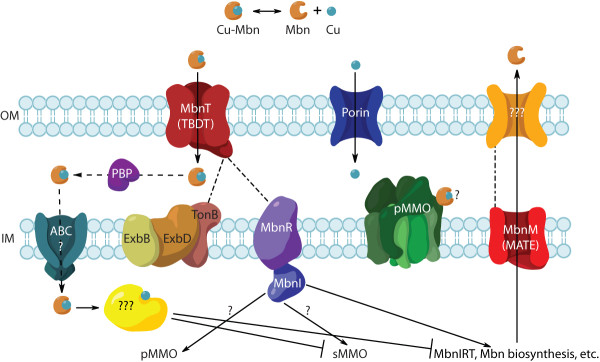
**Proposed Mbn signal transduction pathway featuring the MbnIRT triad**. Mbn is secreted from the cell via the MATE multi-drug exporter MbnM and an unknown outer membrane partner. CuMbn is readmitted to the cell via the TonB-dependent transducer MbnT. CuMbn binding to MbnT induces a conformational change that results in contact with both the inner-membrane TonB-ExbD-ExbB complex and MbnR via the unique N-terminal extension of MbnT. CuMbn may or may not enter the cytoplasm intact, but either way, MbnR activates MbnI analogous to a standard FecIRA system. MbnI replaces σ^70 ^in the active RNA polymerase complex, activating transcription of Mbn biosynthesis and transport genes, and potentially other operons needed in low copper conditions. In siderophore systems, the negative regulator Fur binds iron as intracellular iron levels rise, and the holo Fur binds to siderophore biosythesis and transport promoter regions, inhibiting transcription. A similar negative regulator might be needed to trigger the copper switch to pMMO production.

Strikingly, only the four *Methylosinus *MbnT TBDTs have the N-terminal extensions necessary for FecIRA signaling [57], suggesting a possible regulatory mechanism for Mbn production and transport in Group I operons (Figure 6). In this model, when CuMbn binds to MbnT, a periplasmic TonB-mediated interaction with MbnR results in an altered cytoplasm-side interaction with MbnI. The MbnI ECF sigma factor may then interact with RNA polymerase to either upregulate or inhibit Mbn biosynthesis and transport and may also regulate other operons that are highly expressed at low copper, such as the sMMO operon. If MbnIRT is a positive regulation system, a negative regulator that binds copper and represses Mbn biosynthesis and transport, among other systems, may also be present.

The TBDTs in other operons beyond the *Methylosinus *(Group I) species lack N-terminal extension domains and are not adjacent to FecIR homologues. Although a FecIRA-like system could still be present in these species in a distant small operon, it is less likely. It may be that models analogous to different siderophore regulatory systems are more relevant to these Mbn operons. For example, iron-loaded pyochelin is taken up into the cell and binds to the transcription factor PchR, which regulates its biosynthesis and transport [66-69]. If such a system exists for chalkophores (Additional file 1, Figure S5), the regulators do not appear to be consistently encoded near the biosynthesis operon. However, genes encoding periplasmic binding proteins, commonly associated with natural product import via adenosine triphosphate (ATP)-binding cassette (ABC) transporters, are located downstream of TBDTs in both complete *Methylocystis *and *Azospirillum *operons, and could be relevant to the need for cytoplasmic uptake in a PchR-like model (Figure 2).

### MbnP and MbnH: mysterious partners

The genes encoding MbnP and MbnH are conserved as a pair far beyond the group of Mbn producers analyzed here and are defined by an existing set of TIGRFAM HMMs (TIGR04039 and TIGR04052) and an associated genome property (GenProp0940). The pair consists of the di-heme cytochrome *c *peroxidase MbnH, frequently annotated as resembling MauG, and its neighboring partner protein, MbnP. In two non-Group V genomes (*Methylosinus *sp. LW3 and LW4), there are cases where this pair is not immediately proximal to an Mbn operon, but is present elsewhere in the genome. *Methylosinus trichosporium *OB3b has two such additional pairs. Interestingly, these isolated pairs are located near MbnT-like TBDTs that also have adjacent MbnI and MbnR homologues.

A somewhat similar pair of proteins are found in some methanotroph species that lack Mbn operons. In *Methylococcus capsulatus *(Bath), the proteins are called SACCP (the di-heme cytochrome *c *peroxidase) and MopE (the partner protein). MopE is known to be the subject of a post-translational modification (possibly by SACCP, which is similar to MauG [70]) in which a tryptophan converted to kynurenine participates in a copper binding site [71]. Additionally, while the intact MopE protein is surface-associated, a C-terminal region is fully secreted [72]. In *Methylomicrobium album *BG8, these proteins are called CorB (the di-heme cytochrome *c *peroxidase) and CorA (the partner protein) [73,74]. The genes encoding these proteins are downregulated in the presence of copper [75-77]. However, although there are several well-conserved tryptophans in the MbnP proteins, the sequence is not markedly similar to MopE or CorA (Additional file 1, Figure S6), and there are no data linking any close MbnP homologues or their di-heme cytochrome *c *peroxidase partners to copper. The relevance of this gene pair to Mbn biosynthesis, regulation or transport thus remains unclear.

### Overall structure of the Mbn operon

The core of the Mbn operon (Figure 2) is the *MbnB *biosynthesis gene, located directly downstream of *MbnA *in all operons except for the two Group V operons, which have an unknown gene between *MbnA *and *MbnB. MbnC *encodes a secondary core protein, present immediately downstream of *MbnB *in all operons except Group V operons. All components beyond that core are more flexible. When present, *MbnM *follows the core biosynthesis peptides. Other biosynthesis-related genes, such as *MbnN *and *MbnS *follow MbnM. In some cases, the *MbnP/MbnH *pair appears after the biosynthesis proteins. In others, it is present before them on the same strand, or before them but on the complementary strand. *MbnT*, downstream of *MbnI/R *in Group I operons, primarily occurs prior to the biosynthesis cluster on the same strand and frequently neighbors the *MbnP*/*MbnH *pair as well.

In many of the operons, factors related to genetic mobility, such as insertion sequences, transposases, integrases, insertion sites, shufflons and conjugation-related proteins, occur on one or both sides of the Mbn operon or within several kilobases (Figure 2). These elements may suggest an explanation for the seemingly unrelated assortment of species in which these operons have been detected, and for the lack of operon detection in several well-studied methanotroph species, including *Methylocystis *str. Rockwell [78]. Siderophores are sometimes transported between species on virulence or fitness cassettes [79]. Similarly, it may be that chalkophores are transported in this fashion and adapted by species that have a special need for copper-binding compounds.

## Conclusions

We have detected a total of 18 novel Mbn-like precursors located in full or partial biosynthesis/transport operons in 16 species or metagenomic samples. Of the methanotroph species, operons are present in both strains that undergo the copper switch from sMMO to pMMO (for example, *Methylosinus trichosporium *OB3b [28], *Methylocystis *str. M [80,81], *Methylocystis hirsuta *CSC-1 [82]) and those that only express pMMO (for example, *Methylocystis parvus *OBBP [40], *Methylocystis rosea *SV97T [83]). The 16 species are not limited to methanotrophic bacteria, providing compelling evidence that Mbn-like compounds may play a broader role in proteobacterial metal homeostasis. This analysis reveals the precursor peptide for *Methylocystis rosea *SV97T Mbn [24] and identifies in the same operon genes encoding enzymes that would be necessary to produce the novel features of this Mbn, specifically the sulfonated threonine. Moreover, these data allow us to predict that the Mbn produced by *Methylocystis *strain SC2 will be very similar to that of *Methylocystis rosea *SV97T and likely identical to that of *Methylocystis hirsuta *CSC-1. Conversely, we can predict that the Mbn operons of *Methylocystis *str. SB2, *Methylocystis *str. M and *Methylocystis hirsuta *CSC-1 will have the same core components as the two *Methylocystis *operons presented here. Taken together, these findings provide strong new support for a post-translational modification biosynthetic pathway.

Beyond the four *Methylocystis *Mbns, the only other structurally characterized Mbn is the original compound from *Methylosinus trichosporium *OB3b, which has a Group I Mbn operon. As the related natural products from Group I, III, IV and V familes are characterized, the extent of structural diversity in the Mbn family should become more clear. The roles of MbnB and MbnC as well as the less universal MbnN, MbnS and MbnF proteins in biosynthesis are unknown or unconfirmed and need to be investigated biochemically. This is particularly important since Mbns contain uncommon post-translational modifications, such as thioamide groups, a modification rare enough that Mbns have doubled the number of compounds known to contain it [84]. In addition, there are no other examples of RiPPs containing pyrazinediones [85,86], and even oxazol*one *rings are uncommon, with oxazoles and thiazoles constituting the more common products of serine, threonine and cysteine cyclization. The combination of these motifs with the possibility of more unknown post-translational modifications in Mbns from Groups I and III to V suggests that novel biochemical mechanisms may be involved in Mbn biosynthesis.

The two identified Group V operons may represent a different natural product subfamily, albeit one that shares some similar biosynthesis proteins and modifications with the main Mbn family. Notably, their MbnA sequences contain only a single modifiable cysteine, suggesting that if the final products bind copper at all, they do not use the paired heterocycle/thioamide coordination scheme. Instead of MbnC homologues, these operons include a third unidentified putative protein which neighbors *MbnA*, and *Vibrio caribbenthicus *also has a second unknown protein following *MbnB*. Both have nearby exporters, but no TBDT-like importers.

The identification of MbnM and MbnT as common members of the Mbn operon provides candidate transporters for both Mbn import and export. The possible involvement of MATE-type exporters is somewhat surprising, but the ability of TBDTs to import metal-loaded siderophores is well documented, and the association of such transporters with Mbn operons supports experimental work showing that Mbn uptake is an active process [21,53-55]. Furthermore, in the case of Group I operons, the N-terminal transduction element in MbnT combined with the presence of MbnI and MbnR is consistent with FecIRA-style regulation. This model, along with a hypothetical pyochelin-like route for non-Group I operons, provides testable mechanisms for CuMbn involvement in methanotrophic copper regulation, and may help unravel the mystery of the copper switch.

A final point of interest lies in what was not found in this analysis. There are a variety of methanotroph genomes, including but not limited to *Methylococcus capsulatus *(Bath) [87], *Methylocella sylvesteris *BL2 [88], *Methylocystis *str. Rockwell (ATCC 49242) [78] and *Methylomicrobium album *BG8, in which we detect no Mbn biosynthesis/transport operons. Based on their genomes, if these species produce a chalkophore as suggested [89], it is not similar to existing structurally characterized Mbns and its biosynthetic enzymes do not closely resemble MbnB and MbnC. While one of these species only produces sMMO, the rest produce pMMO and some, including *Methylocystis *str. Rockwell, produce only pMMO. If these methanotrophs do not produce their own chalkophores, they might scavenge chalkophores from other species, similar to what is observed for siderophores [90], and may still possess Mbn-transporting TBDTs. Alternatively, these strains may have other, yet to be unidentified, mechanisms of copper uptake. Taken together, these data provide new insight into Mbn and Mbn-like compounds and their biosynthesis, provide new tools for investigating these processes, and have implications for the broader question of bacterial heavy metal homeostasis.

## Methods

### Genomes

The contigs, scaffolds or complete genomes containing Mbn operons discussed in this paper include: *Azospirillum *sp. B506 (GenBank: BADK01001132), *Azospirillum *sp. B510 (GenBank: NC_013854) [91], Bioreactor metagenome PBDCA2 (GenBank: AGTN01410593, AGTN01295401 and AGTN01527378), *Cupriavidus basilensis *B-8 (GenBank: AKXR01001597)*, Gluconacetobacter *sp. SXCC-1 (GenBank: NZ_AFCH01000034) [92]*, Gluconacetobacter oboediens *174bp2 (GenBank: NZ_CADT01000094), Marine metagenome (GenBank: JCVI_SCAF_1096627660232), *Methylocystis *sp. SC2 (GenBank: NC_018485) [43], *Methylobacterium *sp. B34 (GenBank: BADE01000957), *Methylocystis parvus *OBBP (GenBank: AJTV01000003 and AJTV01000041) [40]*, Methylocystis rosea *SV97T (IMG: A3OODRAFT_scaffold1.1)*, Methylosinus *sp. LW3 (IMG: MetLW3DRAFT_contig2.2), *Methylosinus *sp. LW4 (IMG: MetLW4DRAFT_scaffold2.2)*, Methylosinus trichosporium *OB3b (IMG: MettrDRAFT_Contig106; GenBank version is outdated) [28], *Photorhabdus luminescens *subsp. *laumondii *TT01 (GenBank: BX571860) [93], *Pseudomonas extremaustralis *14-3 substr. 14-3b (GenBank: NZ_AHIP01000040) [94], *Pseudomonas fluorescens *NZI7 (GenBank: AJXF01000037) [95]*, Tistrella mobilis *KA081020-065 (GenBank: NC_017956) [96] and *Vibrio caribbenthicus *ATCC BAA-2122 (GenBank: NZ_AEIU01000072).

### Gene cluster identification and classification

Gene sequences from *Methylosinus trichosporium *OB3b and *Azospirillum *sp. B510 were used as seeds for searches against the NCBI WGS and NR databases [97] (National Center for Biotechnology Information, Bethseda, Maryland, USA), as well as the IMG database [98] (DOE Joint Genome Institute, Walnut Creek, California, USA), using the tBLASTn [99] (National Center for Biotechnology Information, Bethseda, Maryland, USA) algorithm to identify genes even in unannotated regions. Hits of E <1E-20 were manually examined for the presence of other related genes and potential precursors. In genes of interest, annotation was confirmed or potential roles for unannotated genes were identified via BLAST (National Center for Biotechnology Information, Bethseda, Maryland, USA) and via the Pfam (European Bioinformatics Institute, Hinxton, England, UK) and TIGRFAM (J. Craig Venter Institute, Rockville, Maryland, USA) databases [38,100]. In the cases of *Cupriavidus basilensis *B-8, *Methylocystis parvus *OBBP, *Pseudomonas fluorescens *NZI7, *Azospirillum *sp. B506 and *Methylobacterium *sp. B34, the relevant contigs were manually examined for ORFs. ORFs of interest were provisionally identified using BLAST and PFAM. The IGS Annotation Engine (http://ae.igs.umaryland.edu/cgi/index.cgi) [101] (Institute for Genome Sciences, University of Maryland, Baltimore, Maryland, USA) was used for structural and functional annotation of the first three of these sequences (Additional files 3, 4, 5, 6). Manatee was used to view annotations (http://manatee.sourceforge.net/).

### Alignment and phylogeny

Initial multiple sequence alignments were generated using ClustalOmega [102] (University College Dublin, Dublin, Ireland), MAFFT [103] (Computational Biology Research Center, Tokyo, Japan) or MUSCLE [104] (Drive5, Tiburon, California, USA), applying the default settings. Alignments were visualized using the Jalview 2 (University of Dundee, Dundee, Scotland, UK) package and were organized according to a simple tree based on the Neighbor-Join algorithm using the BLOSUM62 model. Phylogenetic and evolutionary analyses were conducted using the MEGA5 package [105] (Center for Evolutionary Medicine and Informatics, Arizona State University, Tempe, Arizona, USA). During phylogentic tree construction, the evolutionary history was inferred by using the Maximum Likelihood method based on the JTT matrix-based model [106]. The bootstrap consensus tree inferred from 1,000 replicates [107] was taken to represent the evolutionary history of the taxa analyzed [106]. Branches corresponding to partitions reproduced in less than 50% bootstrap replicates were collapsed. Initial tree(s) for the heuristic search were obtained automatically by applying Neighbor-Join and BioNJ algorithms to a matrix of pairwise distances estimated using a JTT model, and then selecting the topology with superior log likelihood value. A discrete Gamma distribution was used to model evolutionary rate differences among sites (five categories (+*G*, parameter varied by alignment)).

### Precursor gene identification

The *Methylosinus trichosporium *OB3b and *Azospirillum *sp. B510 MbnB sequences were used as described above to identify potential Mbn biosynthesis operons. When such a protein was identified, small ORFs (10 to 50 aa) in a 2 kb region up- and downstream of the gene were analyzed for the presence of cysteines in the last 10 residues, a lysine or arginine preceded by a hydrophobic region located a few residues prior to the cysteine, and multiple lysines and/or arginines within the first 10 residues. Motifs showing the amino acid frequency across the precursor peptide were generated using Jalview 2 [108].

### HMM generation and analysis

HMMs were generated for MbnA (Additional file 7), MbnB (Additional file 8) and MbnC (Additional file 9) using HMMER3 [109] (Howard Hughes Medical Institute Janelia Farm, Ashburn, Virginia, USA). Prior to HMM generation, curated seed alignments (featuring removal of truncated or otherwise unacceptable sequences and trimming of extra domains) were manually generated. HMMs were used to scan the existing protein databases for additional operons, but all extant Mbn operons appear to have been located via tBLASTn, prior to HMM construction. A revised NExT HMM (NexTNew) was used to confirm the identification of TBDTs possessing the N-terminal extension required for TonB-mediated signal transduction [57].

## Abbreviations

Aa: amino acids; ABC: ATP-binding cassette; ATP: adenosine triphosphate; BLAST: Basic Local Alignment Search Tool; ECF: extracytoplasmic function; FAD: flavin adenine dinucleotide; HMM: hidden Markov model; MATE: multidrug and toxic compound extrusion; Mbn: methanobactin; MFS, major facilitator superfamily; MMO: methane monooxygenase; Mrna: messenger ribonucleic acid; NExT: N-terminal extension; NMR: nuclear magnetic resonance; ORF: open reading frame; pMMO: particulate methane monooxygenase; RiPP: ribosomally synthesized and post-translationally modified peptide natural product; RND: resistance-nodulation-cell division; SACCP: surface-associated cytochrome c peroxidase; SAM: S-adenosyl methionine; sMMO: soluble methane monooxygenase; TBDT: tonB-dependent transporter.

## Competing interests

The authors declare that they have no competing interests.

## Authors' contributions

GEK carried out the bioinformatics analyses. GEK and ACR conceived of the study and wrote the manuscript. Both authors read and approved the final manuscript.

## Supplementary Material

Additional file 1 **Figures S1-S6**. Figures of additional Mbn structures, alignment of potential biosynthesis protein in Group V operons, alignment of MbnM sequences, alignment of MbnT sequences and phylogenetic tree, figure of alternate Mbn regulation scheme, and alignment of MbnP sequences. Click here for file

Additional file 2 **Table S1**. Table containing information regarding the GenBank or JGI contigs/scaffolds and locus IDs (where present) for the genes discussed in the paper. Click here for file

Additional file 3 **Annotated *C. basilensis *B-8 scaffold 1267_1, GenBank format**. A GenBank-formatted file consisting of *C. basilensis *B-8 scaffold 1267_1 (containing an Mbn operon), as annotated by the IGS. Click here for file

Additional file 4 **Annotated *M. parvus *OBBP contig 003, GenBank format**. A GenBank-formatted file consisting of *M. parvus *OBBP contig003 (containing an Mbn operon), as annotated by the IGS. Click here for file

Additional file 5 **Annotated *M. parvus *OBBP contig 041, GenBank format**. A GenBank-formatted file consisting of *M. parvus *OBBP contig041 (containing an Mbn operon), as annotated by the IGS. Click here for file

Additional file 6 **Annotated *P. fluorescens *NZI7 contig00040_contig01, GenBank format**. A GenBank-formatted file consisting of *P. fluorescens *NZI7 contig00040_contig01 (containing an Mbn operon), as annotated by the IGS. Click here for file

Additional file 7 **HMMER3 hidden Markov model for MbnA**. A HMMER3-compatible hidden Markov model constructed using a curated alignment of the MbnA sequences discussed in the paper. Click here for file

Additional file 8 **HMMER3 hidden Markov model for MbnB**. A HMMER3-compatible hidden Markov model constructed using a curated alignment of the complete MbnB sequences discussed in the paper. Click here for file

Additional file 9 **HMMER3 hidden Markov model for MbnC**. A HMMER3-compatible hidden Markov model constructed using a curated alignment of the MbnC sequences discussed in the paper. Click here for file
