# Supplementary material for: Genome mining for methanobactins
Source: BMC Biol. 2013 Feb 26;11:17. doi: 10.1186/1741-7007-11-17 (PMC3621798; doi:10.1186/1741-7007-11-17)
Supplement: Additional file 1 — Figures S1-S6. Figures of additional Mbn structures, alignment of potential biosynthesis protein in Group V operons, alignment of MbnM sequences, alignment of MbnT sequences and phylogenetic tree, figure of alternate Mbn regulation scheme, and alignment of MbnP sequences. [file 1741-7007-11-17-S1.PDF]

Supporting Information for

Genome mining for methanobactins

Grace E. Kenney and Amy C. Rosenzweig*

Departments of Molecular Biosciences and of Chemistry, Northwestern University, Evanston, Illinois, 60208, USA

*Corresponding author

Email addresses:

GEK: gkenney@u.northwestern.edu

ACR: amyr@northwestern.edu

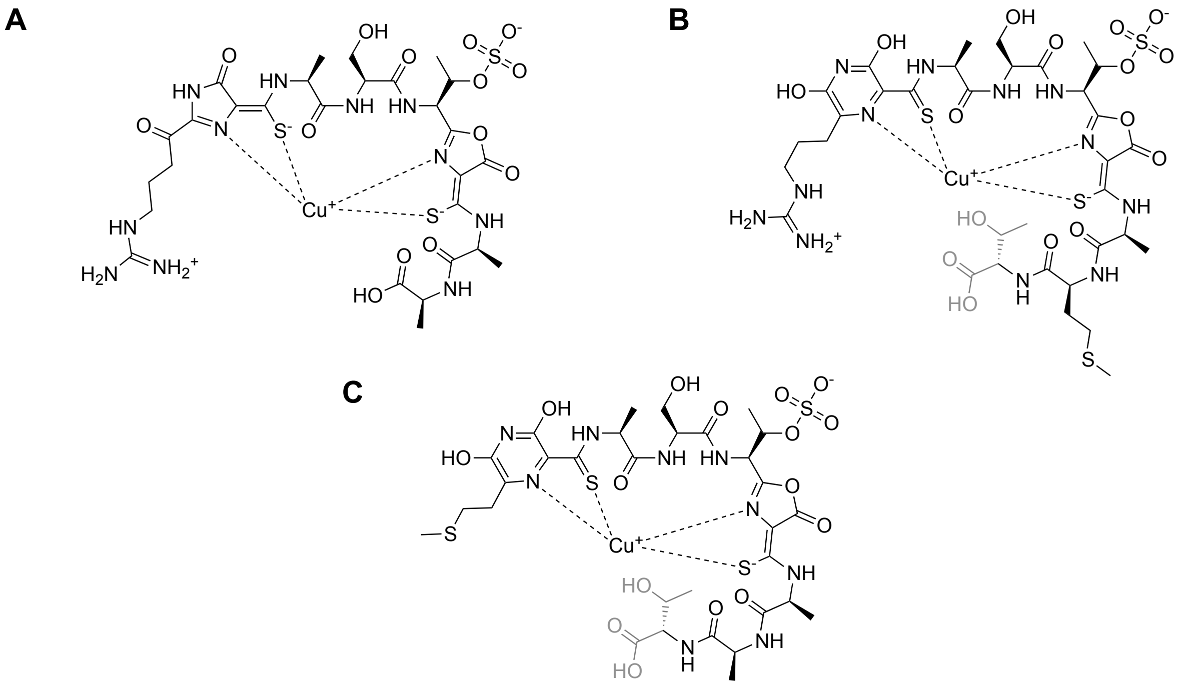

Figure S1 Additional structurally characterized methanobactins. (A) Structure of Mbn from *Methylocystis* strain SB2 determined by NMR. (B) Structure of Mbn from *Methylocystis* strain M determined by X-ray crystallography. This structure is known to be missing at least one residue, a C-terminal threonine. (C) Structure of Mbn from *M. hirsuta* CSC-1 determined by X-ray crystallography. This structure is known to be missing at least one C-terminal threonine.

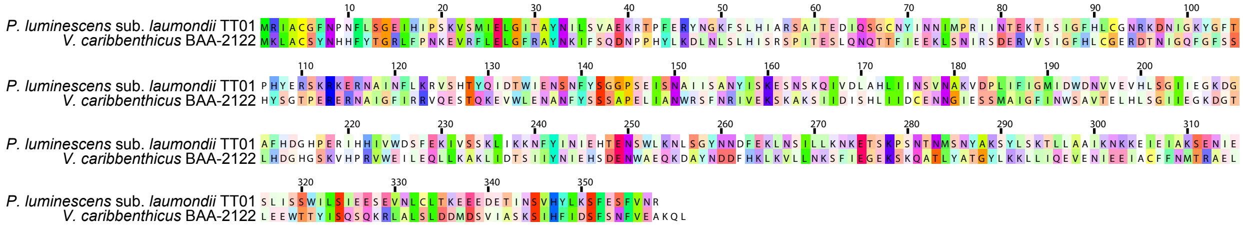

Figure S2 Group V Mbns have a potential biosynthesis protein that is not MbnC. MUSCLE alignment of the two ORFs that are located between the MbnA and MbnB sequences in *V. caribbenthicus* BAA-2122 and *P. luminescens* TTO1. These ORFs show weak similarity to the DUF692 family (of which MbnB is a member) and no strong homology to any ORFs from other genomes.

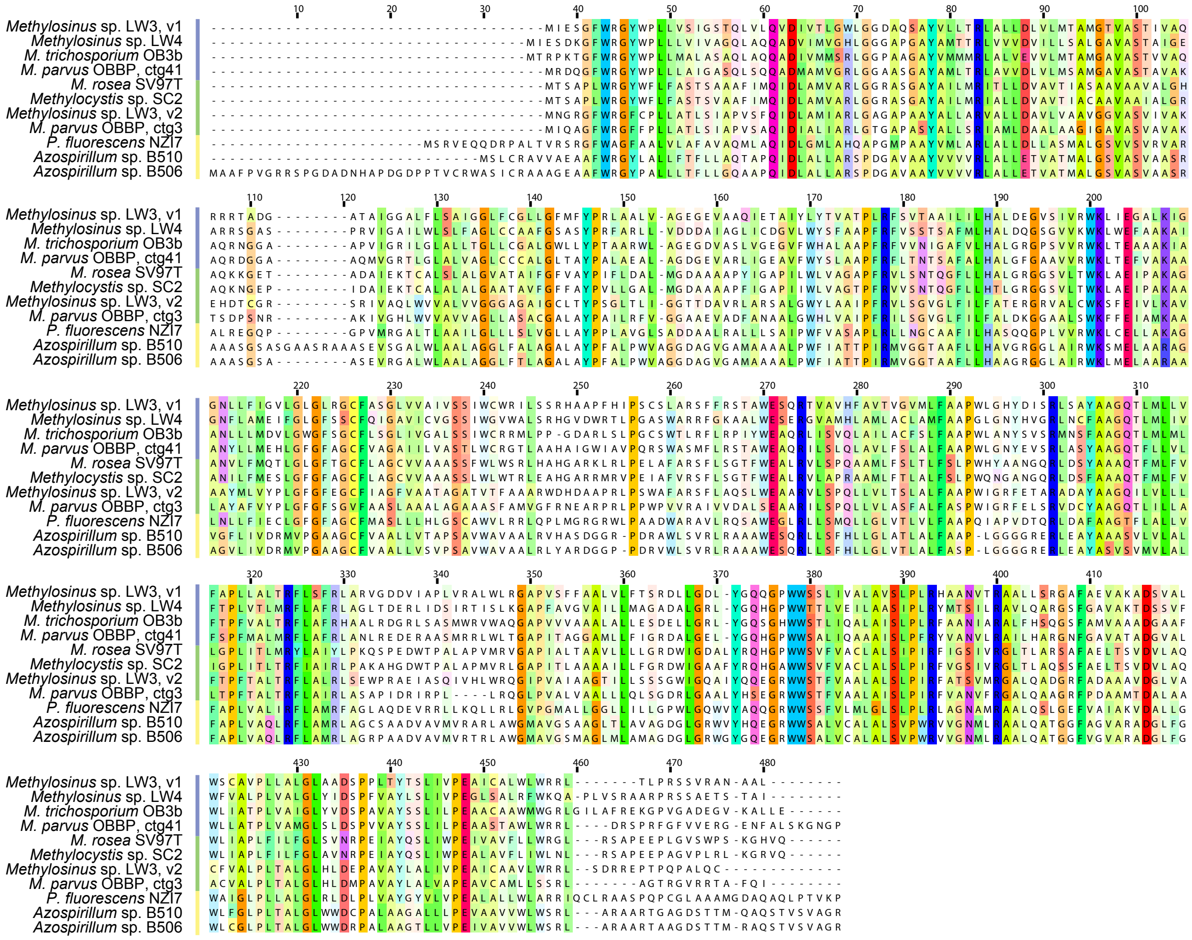

Figure S3 Sequence alignment (MUSCLE) of MbnM, the possible Mbn exporter. The MATE exporters from the two Group V Mbn operons are somewhat divergent, and are not shown. Residues are colored following the Taylor color scheme [98], with intensity modified by a conservation color increment of 30%. Truncated metagenomic sequences are not shown in this alignment.

A

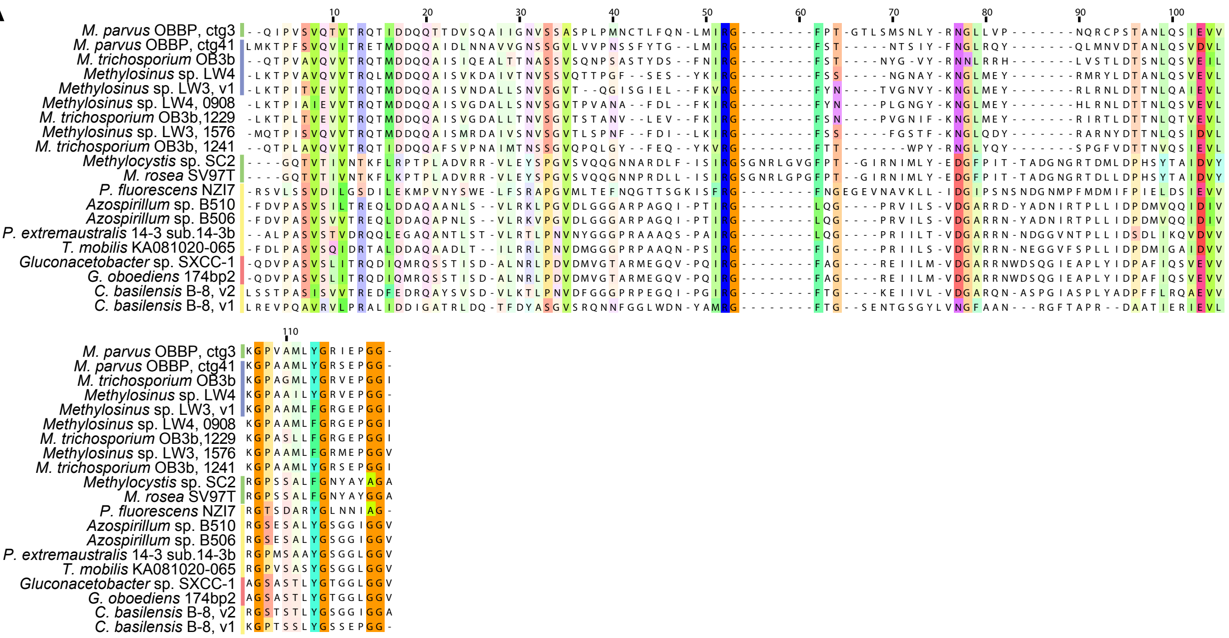
M. parvus OBBP, ctg3
M. parvus OBBP, ctg41
M. trichosporium OB3b
Methylosinus sp. LW4
Methylosinus sp. LW3, v1
Methylosinus sp. LW4, 0908
M. trichosporium OB3b,1229
Methylosinus sp. LW3, 1576
M. trichosporium OB3b, 1241
Methylocystis sp. SC2
M. rosea SV97T
P. fluorescens NZI7
Azospirillum sp. B510
Azospirillum sp. B506
P. extremaustralis 14-3 sub.14-3b
T. mobilis KA081020-065
Gluconacetobacter sp. SXCC-1
G. oboediens 174bp2
C. basilensis B-8, v2
C. basilensis B-8, v1

B

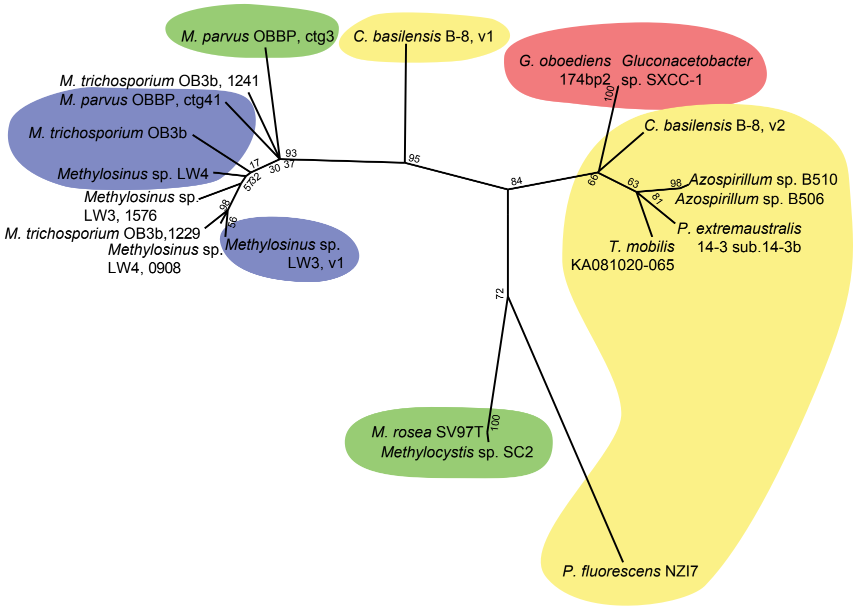
M. parvus OBBP, ctg3
C. basilensis B-8, v1
G. oboediens 174bp2
Gluconacetobacter sp. SXCC-1
M. trichosporium OB3b, 1241
M. parvus OBBP, ctg41
M. trichosporium OB3b
Methylosinus sp. LW4
Methylosinus sp. LW3, 1576
M. trichosporium OB3b,1229
Methylosinus sp. LW4, 0908
Methylosinus sp. LW3, v1
C. basilensis B-8, v2
Azospirillum sp. B510
Azospirillum sp. B506
P. extremaustralis 14-3 sub.14-3b
T. mobilis KA081020-065
M. rosea SV97T
Methylocystis sp. SC2
P. fluorescens NZI7

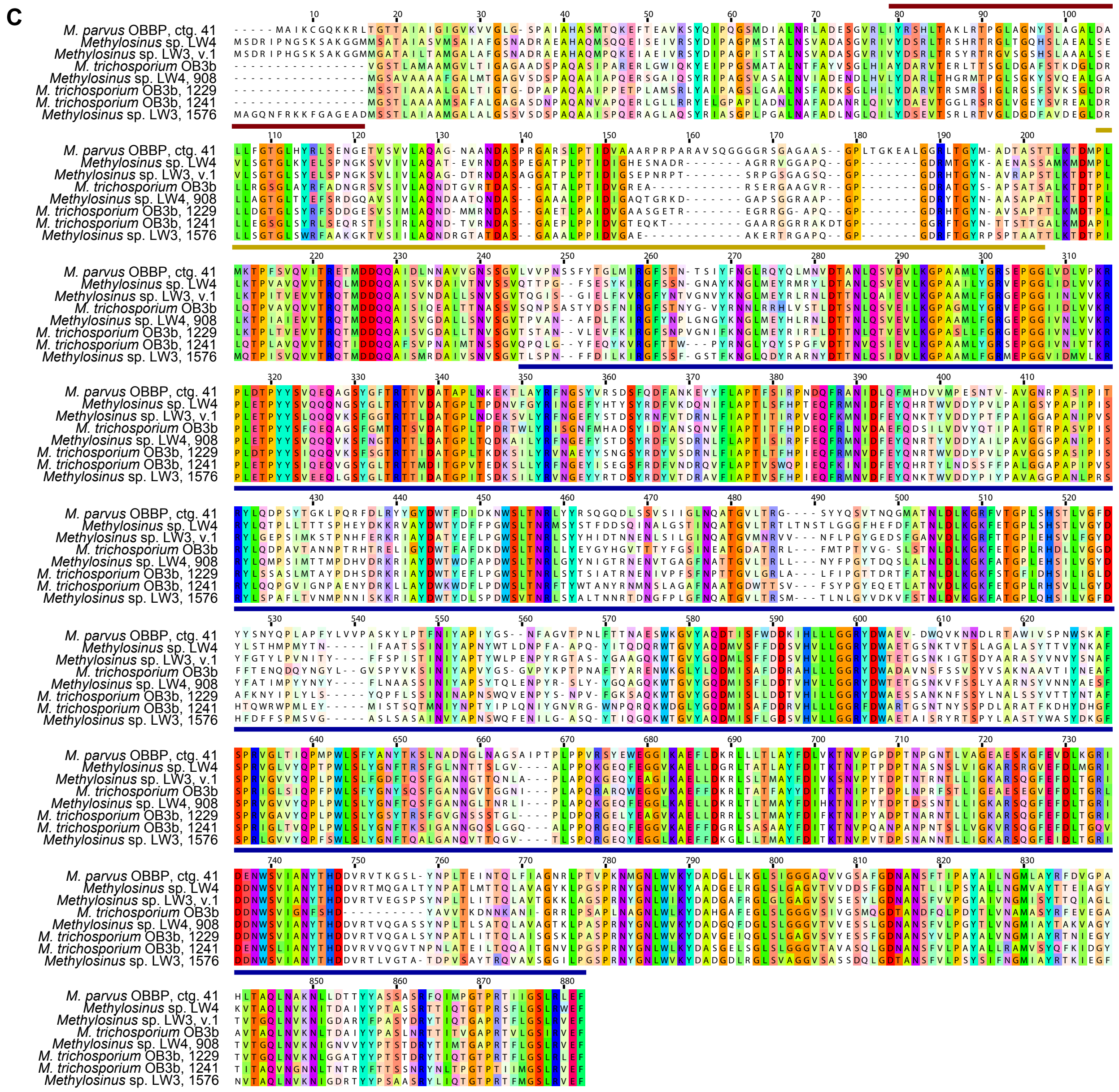

Figure S4 Sequence analysis of MbnT. (A) MUSCLE alignment of MbnT plug domains from all Mbn operons (as well as the non-operon MbnIRTPH loci found in the three *Methylosinus* species). (B) Phylogenetic tree (constructed in MEGA 5.0 as described in the Methods section) based on a MUSCLE alignment of MbnT plug domains. Gamma distribution parameter 3.1209. The similarity between the Group I and Group II plug domains is much less than might be expected on the basis of gene homology in the rest of the operon. This may reflect the effect of

divergent backbone sequences and three-dimensional structures on substrate recognition by TBDT plug domains. (C) MUSCLE alignment of Group I MbnT sequences, including the non-operon MbnIRTPH loci found in the three *Methylosinus* species (indicated by the addition of their locus IDs to their labels.) The N-terminal extension, present in Group I Mbn operons only, is highlighted with a dark red line. The plug domain is highlighted with a gold line, and the main ß-barrel is highlighted with a blue line.

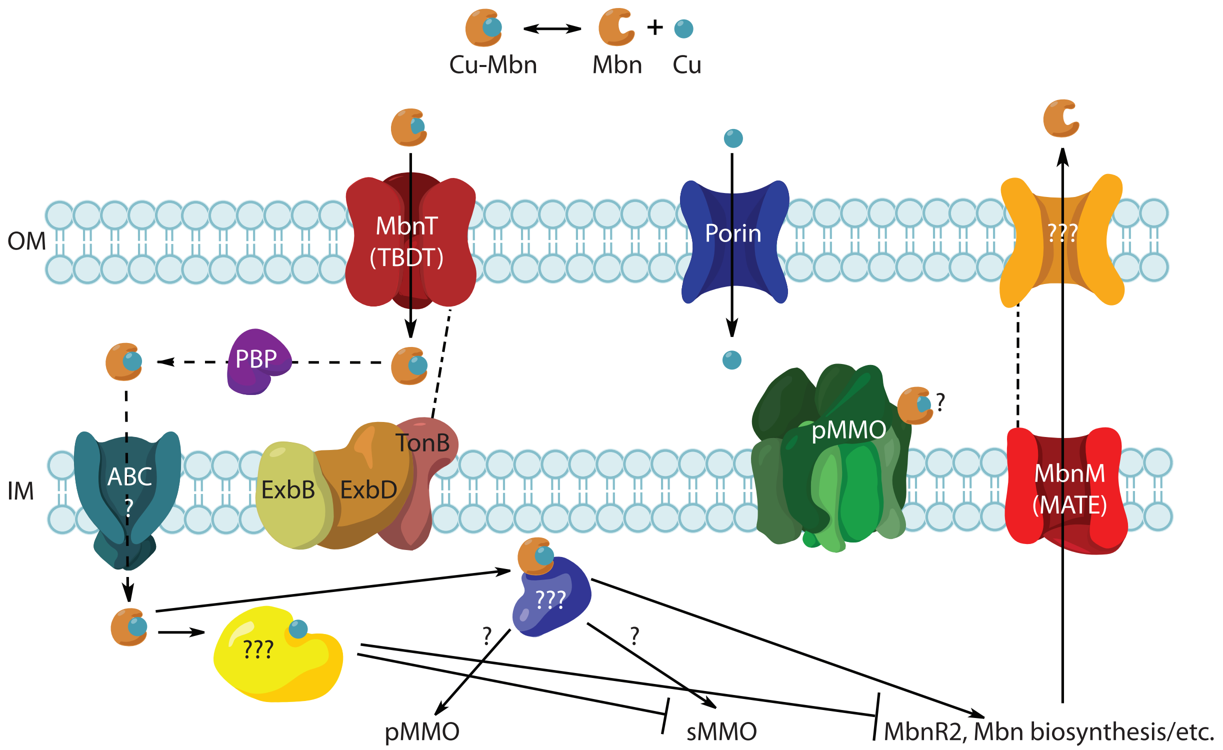

Figure S5 Alternate Mbn regulation scheme employing a PchR-like positive regulator and no transducing TBDTs. Unlike pyoverdine, which has a FecARI-style regulation system involving transmembrane signal transduction, pyochelin follows a different model. In this model, the holo siderophore is imported into the cytoplasm via an ABC transporter. PchR binds the holo siderophore specifically and serves as an activator for siderophore biosynthesis and related activities. In species without an MbnIRT signal transduction system, a different regulatory system would be required. A pyochelin-like system, employing an ABC transporter to internalize CuMbn and a CuMbn-binding regulatory factor, is one possibility. In this model, a negative metal-binding regulator, analogous to Fur, might also be expected.

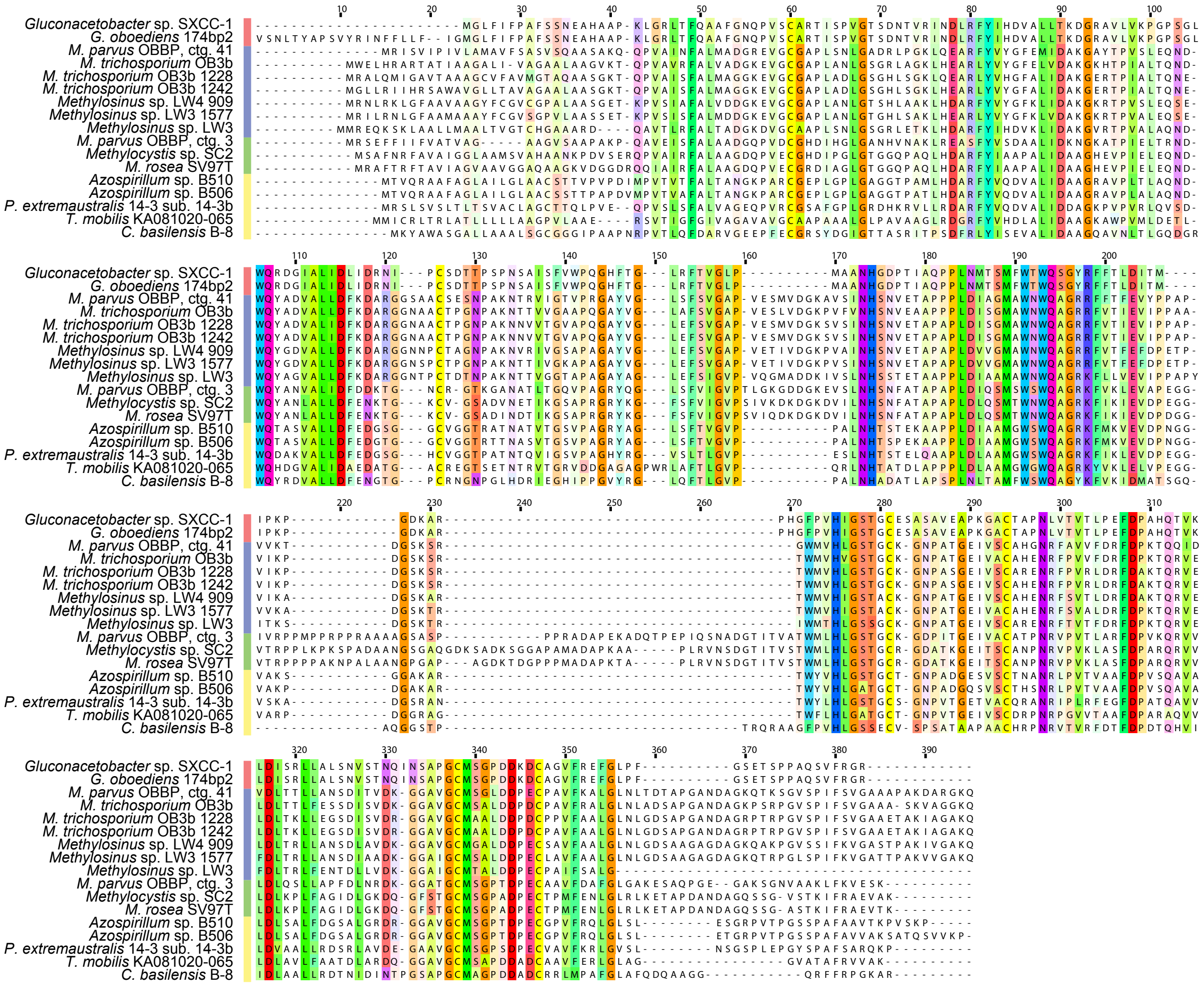

Supplemental Figure 6 Sequence alignments of MbnP. These MUSCLE alignments including sequences from the extra MbnIRTPH loci present in several Group I genomes. Several strongly conserved tryptophans exist in MbnP, as well as a well-conserved histidine, but they are not in positions that closely resemble those of their potential counterparts in MopE and CorB.
